# *Leishmania infantum* Specific Humoral and Cellular Immune Responses in Cats and Dogs: A Comparative Cross-Sectional Study

**DOI:** 10.3390/vetsci9090482

**Published:** 2022-09-07

**Authors:** Vito Priolo, Pamela Martínez-Orellana, Maria Grazia Pennisi, Ana Isabel Raya-Bermúdez, Estefania Jurado-Tarifa, Marisa Masucci, Giulia Donato, Federica Bruno, Germano Castelli, Laia Solano-Gallego

**Affiliations:** 1 Dipartimento di Scienze Veterinarie, Università di Messina, 98168 Messina, Italy; 2 Departament de Medicina i Cirurgia Animals, Facultat de Veterinaria, Universitat Autònoma de Barcelona, 08193 Bellaterra, Spain; 3 Facultad de Medicina Veterinaria, Universidad de Córdoba, Campus Rabanales, 14014 Córdoba, Spain; 4 Centro di Referenza Nazionale per la Leishmaniosi (CReNaL), Istituto Zooprofilattico Sperimentale della Sicilia “A. Mirri”, 90129 Palermo, Italy

**Keywords:** leishmaniosis, feline, canine, IFN-γ, whole blood assay, PCR, feline immunodeficiency virus, prevalence, ELISA, IFAT

## Abstract

**Simple Summary:**

The role of the cat as a reservoir of *Leishmania infantum* and the characteristics of the immune response to this infection remains limited, contrary to the dog. This study aimed to compare the rate of *L. infantum* infection and parasite-specific humoral and cell-mediated immune responses in cats and dogs living in an area endemic to canine leishmaniosis (Córdoba, Spain). About one-third of the dogs and cats studied were positive for at least one molecular or serological diagnostic test. The immunopathogenesis of *L. infantum* infection in cats showed similarities when compared to dogs, although the parasite-specific immune response level in dogs was generally higher than in cats. This study shows that stray cats are exposed to *L. infantum* infection similarly to dogs in endemic areas, are able to mount a specific anti*-Leishmania* humoral and cell-mediated immune response as dogs, and can contribute to the endemicity of infection.

**Abstract:**

Dogs are the main reservoir of *Leishmania infantum* and display different immunological patterns correlating with the progression of infection to disease. Data about feline *L. infantum* adaptive immune response are scant. This study aimed to compare the prevalence and immune response in cats and dogs from the same endemic area of canine leishmaniosis. Stray cats (109) and rescued dogs (59) from Córdoba (Spain) were enrolled. Data about their exposure to *L. infantum* were analyzed by detection of parasite DNA, measurements of *Leishmania-*specific interferon-γ (whole blood assay in 57 cats and 29 dogs), and antibodies (enzyme-linked immunosorbent assay and immunofluorescence antibody test). An overall *L. infantum* prevalence of 30.5% in dogs and 30% in cats were found according to serology and PCR tests. Prevalence was 44.8% in dogs and 35.1% in cats tested also for interferon-γ production. Dogs showed higher anti-*L. infantum* antibody levels compared to cats. More than one-third of cats had contact with or were infected by *L. infantum* and they may contribute to the endemicity of leishmaniosis in the investigated region. The immunopathogenesis of feline *L. infantum* infection has similarities with dogs but cats show a lower level of adaptive immune response compared to dogs.

## 1. Introduction

*Leishmania infantum* is the most common species of the *Leishmania* genus in Europe, responsible for a neglected vector-borne zoonosis [1]. Vectorial transmission is carried by the bite of female sand flies of the genus *Phlebotomus* and dogs are considered the principal reservoir of this infection [2]. In many regions of southern Europe, *L. infantum (Li)* is endemic with a reported prevalence of dogs with anti-*Leishmania* antibodies ranging from 0 to more than 50%. Higher rates of positivity have been reported in studies where the parasite DNA was investigated in various canine tissues by polymerase chain reaction (PCR) [3]. Other domestic and wild animals are also infected such as cats [4,5], horses [6], and hares [7,8,9]. The role of the domestic cat as a reservoir of infection remains unclear. However, in endemic areas, the prevalence of cats with anti-*Leishmania* antibodies has been found to range between 0 and 68.5% [2,4,10,11,12,13], and blood PCR prevalence between 0 and 60.6% [2,4,11,12,13,14]. Furthermore, it is proven that cats are exposed to the bite of sand flies and, after the blood meal on an infected cat, females become infected and are able to transmit the infection to dogs [15,16,17]. Dogs display a wide range of immunological patterns that correlate with the progression of infection and severity of disease [18]. Briefly, in dogs, CD4+ Th1 lymphocytes activate macrophages to effector cells, by producing *Leishmania*-specific interferon-γ (IFN-γ). This immune response enables the host to fight the parasite and control the progression of the disease [19]. Conversely, a reduced or absent cell-mediated immune response is predominant in dogs with clinical disease and poor clinical outcomes [20,21,22,23,24,25]. It was recently demonstrated that both parasite-specific cell-mediated and humoral adaptive immune responses are mounted in cats [4,23,26,27]. In particular, we observed parasite-specific IFN-γ production in 18% of cats, and 78% of them were found negative for *L. infantum* antibody and parasite DNA detection in blood [4]. Therefore, the investigation of IFN-γ production enables us to better estimate the rate of *L. infantum* exposure in cats [4]. There are limited studies that compared *L. infantum* rates of infection or exposure in dogs and cats in the same period and area [13,28]. According to these studies based only on serological or molecular tests, cats appear to have lower rates of exposure and infection than dogs [13,28]. The main goal of this cross-sectional prospective field study was to compare parasite-specific humoral and cell-mediated immune responses, as well as the prevalence of exposure and infection in cats and dogs living in the same area endemic for canine leishmaniosis, combining the results of immunological tests and PCR.

## 2. Materials and Methods

### 2.1. Study Location, Period of Sampling and Animal Enrollment

From May to June 2017, 168 rescued and stray pets (109 stray cats and 59 rescued dogs) from the province of Córdoba (Spain) were enrolled. Stray cats were included during a trap-neuter-release program organized by Córdoba City Council and examined by veterinarians before the elective surgery. Dogs were enrolled in three different kennels located in the suburban area of Córdoba during the annual check-up program for leishmaniosis. Inclusion criteria for enrollment aimed to study dogs and cats at risk of exposure to sand fly bites focusing on animal age, outdoor lifestyle, and treatment against sand fly bites. Therefore, we studied only adults, exposed to at least one (cats) or two (dogs) sand fly seasons, and they had not been treated with ectoparasiticides effective against sand fly bite at least in the last two years. Moreover, as they were kennel dogs and stray cats, all enrolled animals shared an outdoor lifestyle.

### 2.2. Sample Collection, Blood Cell Count and Blood Smear Evaluation

Blood was aseptically collected and put into heparin tubes (1 mL for cats and 3 mL for dogs) for IFN-γ release whole blood assay (WBA) and EDTA tubes (1 mL) for complete cell blood count (CBC) (29 dogs and 57 cats) and *L. infantum* PCR. Blood smears were immediately made and afterward stained by May Grünwald-Giemsa stain (Merck KgaA, Darmstadt, Germany) and evaluated microscopically at oil immersion ×1000 magnification for the detection of hemoparasites including *Li* amastigotes [29]. The residual blood was put into empty tubes and centrifuged after clotting at 2000× *g* for 10 min to obtain serum for serological investigations (anti-*L. infantum* antibodies and in cats also anti-FIV antibodies). The WBA and CBC were performed within 24 h after sampling. EDTA blood was stored at +4 °C until used and was brought at room temperature before analyzing. Blood serum and residual EDTA blood were stored at −20 °C until processed for serological (see Section 2.5 and Section 2.6) and PCR investigations (see Section 2.7). The CBC was performed using IDEXX LaserCyte Hematology Analyzer (IDEXX, Westbrook, ME, USA) and manufacturer’s reference interval was considered [30].

### 2.3. Clinical Evaluation

A full clinical examination was performed by veterinarians on 57 cats and 29 dogs tested for WBA. Data about body condition score (BCS = 5/5) [31,32], muscle condition score (MCS = 4/4) [33,34], mucous membranes, skin and lymph nodes were registered in a clinical form. The health status was graded as “very good” when no abnormalities were detected; “good” when minor abnormalities were observed (e.g., mild dermatitis, mild anemia, mild gingivitis); “poor” when 1–2 relevant abnormalities were reported (e.g., cachexia, icterus, moderate to severe anemia, leukocytosis, leukopenia, thrombocytopenia); “very poor” when ≥3 of the latter clinical alterations occurred. Moreover, presence of physical and hematological changes compatible with canine and feline leishmaniosis as BCS ≤ 2/5, MCS ≥ 2/4, lymph node enlargement, skin (ulcerative, nodular, scaly dermatitis), mucosal (nodules, ulcerations) or eye (uveitis) lesions, stomatitis in cats, and non-regenerative anemia were recorded [1,35]. Animals were considered “suspected” of leishmaniosis if they presented ≥1 compatible clinical abnormalities, otherwise they were considered “non suspected”.

### 2.4. Feline and Canine IFN-γ- Release Whole Blood Assays and Sandwich ELISAs

The WBA was performed in dogs (n = 29) and cats (n = 57) as previously described [4,23]. Three different conditions were established: (1) medium alone (2) medium with soluble *L. infantum* antigens (LSA) and (3) medium with mitogen concanavalin A (ConA) (100 mg Medicago * Uppsala, Sweden). Concentrations of IFN-γ were determined using canine and feline commercial sandwich enzyme-linked immunosorbent assay (ELISA) for IFN-γ (DuoSet ELISA, Development Systems R&D, Abingdon, UK) as previously described [4,23]. According to results after stimulation with LSA and ConA, cats [4] and dogs [23], were classified as IFN-γ producers (LSA-IFN-γ-p and ConA-IFN-γ-p) or as IFN-γ non-producers (LSA-IFN-γ-np and ConA-IFN-γ-np).

### 2.5. Serum Antibody Detection against L. Infantum Antigen

Serum antibodies against *L. infantum* antigen were evaluated by both immunofluorescence antibody test (IFAT) and ELISA. A *Leishmania* IFAT was performed as previously described for dogs [35,36] and cats [4]. The cut-off dilution for positivity was set at 1:80 for cats [4], and 1:160 for dogs [36,37]. The endpoint titer of positive samples was determined preparing serial two-fold dilutions of serum. Fluorescence microscope reading was always made by the same operator (MM). The ELISA was performed as previously described for dogs [38] and cats [4]. All samples were analyzed in duplicate, and any plates included a positive (calibrator) and negative control serum from a sick animal and animals from areas where leishmaniosis was not endemic, respectively. The reaction was quantified as ELISA units (EU) related to positive cat and dog sera used as calibrators and arbitrarily set at 100 EU. The cut-off was established at 12.3 EU for cats and at 35 EU for dogs.

### 2.6. Serum Anti-FIV Antibody Detection

A commercial ELISA (Ingenasa^®^, INGEZIM FIV, Madrid, Spain) was used and the manufacturer instructions were followed. A ThermoScientific Multiskan FC spectrophotometer was used for optical density readings.

### 2.7. Leishmania Infantum DNA Extraction and Real Time PCR

Total DNA was extracted from EDTA whole blood using the DNA Gene extraction kit (Sigma Aldrich, St. Louis, MI, USA) following the manufacturer’s instructions with the following modification: 40 μL of proteinase K solution and 400 μL of whole blood were used for all extractions. DNA from the reference *L. infantum* strain MHOM/TN/80/IPT1 was used as a positive control. Whole blood-extracted DNA obtained from clinically healthy dogs and cats from an Italian non-endemic area (mountain village located in the Aosta Valley) that were negative by serological, parasitological, and molecular methods was used as a negative control. The PCR test was targeted at the constant region in the minicircle Kinetoplast DNA (NCBI accession number AF291093). Real Time PCR was developed by the CFX96 Real-time System (Bio-Rad Laboratories s.r.l. Hercules, CA, USA) using TaqMan Master Mix (Applied Biosystems by ThermoFisher). The procedure was performed as previously described [39].

### 2.8. Statistical Analysis

Statistical analysis was conducted using GraphPad Prism version 7.0 for Windows (GraphPad Software, San Diego, CA, USA) and STATA software (StataCorp. 2016. Stata Statistical Software: version 16.1. StataCorp LP: College Station, TX, USA). After confirming the non-normality of the data through the Skewness/Kurtosis test, continuous data were reported as median value, range, 25° and 75° percentile and categorical data were expressed as frequencies. Comparison between the distribution of studied variables (sex, age group, breeds of dogs, rates of positivity at each diagnostic test and overall positivity), between cats and dogs was conducted using Fisher’s Exact test for categorical variables. In animals tested for IFN-γ- release whole blood assays (29 dogs and 57 cats) clinical status, and presence of clinical signs or hematology abnormalities compatible with leishmaniosis were also investigated using Fisher’s Exact test. Statistical analysis of the clinical status was performed comparing two groups of animals: those in “very good” and “good” health status versus the animals in “poor” and “very poor” conditions. Mann–Whitney’s U-test was used to compare unmatched continuous variables (antibody level detected by ELISA or IFAT, parasite load detected by PCR, LSA and ConA-IFN-γ concentrations) between cats and dogs. Wilcoxon’s signed-rank test was used to compare paired continuous variables (LSA and ConA IFN-γ) in the same species. The risk factors for the development of *L. infantum* infection among cats and dogs based on the diagnostic test used were analyzed by using the odds ratio (OR) as a measure of association and the associated 95% confidence interval (CI). Spearman’s correlation coefficient was calculated to evaluate relationships between ELISA, IFAT, and PCR results and between levels of IFN-γ, anti-*Leishmania* antibodies, and *L. infantum* DNA in samples of animals studied. Finally, Cohen’s kappa coefficient was measured to evaluate agreement between IFAT and ELISA.

Differences were considered significant if *p* values were <0.05.

## 3. Results

### 3.1. Cats and Dogs Data

The breed, sex, and age class of both cats and dogs are reported in Table 1. Cats were younger than dogs and a significantly higher number of female cats were included compared to female dogs. Concerning the clinical evaluation of the 57 cats and 29 dogs tested with CBC and WBA, the presence of clinical signs compatible with leishmaniosis was statistically higher in dogs (69%) than in cats (28.1%) (*p* = 0.0004; OR: 5.6944, 95% CI: 2.1456–15.1132). All blood smears of cats and dogs tested with CBC were found negative for *Li* amastigotes and hemoparasites.

### 3.2. Antibody Detection against L. Infantum Antigen

Results of serological tests and their differences among dogs and cats are displayed in Table 2. No differences according to results of serological tests and variables studied (age class, sex, breed) were found in cats and dogs. Ten dogs were positive at both serological tests and a significant correlation was found (*p* = 0.0020; Rho = 0.8988) between ELISA and IFAT where the Cohen’s kappa coefficient (0.708) found a substantial good agreement. No agreement between IFAT and ELISA was detected in cats and correlation was not evaluated considering that only one cat was positive by both techniques. In clinically evaluated dogs and cats, no differences in antibody positivity were found based on their health status and the presence of clinical signs compatible with leishmaniosis.

### 3.3. PCR

Results of PCR tests of dogs and cats are shown in Table 2. The overall median parasite load in blood samples of dogs and cats considered together was 60 parasites/mL (range = 5–440; 25th–75th percentile = 33.75–80.25). No differences according to PCR positivity and variables studied (age class, sex, breed) were found in dogs and cats, respectively. A correlation was not found between PCR results and serological tests (ELISA and IFAT). ELISA or IFAT positive dogs were statistically more frequently PCR positive (ELISA = 5/10, 50%; IFAT = 5/16, 31.2%) than ELISA (2/49, 4.1%; *p* = 0.0009; OR = 23.5; 95% CI = 3.108–127.8) or IFAT negative (2/43, 4.7%; *p* = 0.0126; OR = 9.318; 95% CI = 1.522–49.26) dogs. No statistical difference was found between IFAT-positive cats compared to the negative ones and all PCR-positive cats were ELISA-negative. In clinically evaluated dogs and cats, no differences in PCR positivity were found based on their health status and the presence of clinical signs compatible with leishmaniosis.

### 3.4. LSA-IFN-γ Release Whole Blood Assay

Results of IFN-γ release whole blood assay are shown in Table 2 and Table 3. No statistical differences were found in the number of IFN-γ producer animals according to results of serology (ELISA, IFAT) and PCR in dogs, and cats.

No differences were found in antibody levels (IFAT) between dogs and cats that did not produce IFN-γ after LSA stimulation.

As reported in Table 3, among dogs and cats that produced IFN-γ after LSA stimulation, only one dog (IFAT) and four cats (ELISA, n = 1; IFAT, n = 3) were positive at serology, therefore no comparisons among the antibody levels were performed.

Moreover, no differences in parasite loads were found between IFN-γ producer and non-producer dogs and cats. Data about serological and molecular results of IFN-γ producer and not producer dogs and cats are represented in Table 3. All PCR-positive animals did not produce IFN-γ and three of them (two dogs and one cat) were also positive to at least one serological method. In detail, the cat was IFAT positive with an antibody titer of 1:160 but was negative at ELISA. Conversely, the two dogs were positive on both serological tests (dog 1: 41.3 EU-1:320 IFAT titer; dog 2: 400 EU-1:10,280 IFAT titer).

No differences were found in IFN-γ production after LSA stimulation in dogs and cats according to their health status and the presence of clinical signs compatible with leishmaniosis.

### 3.5. ConA-IFN-γ Release Whole Blood Assay

Results of the ConA-IFN-γ release whole blood assay are listed in Table 2. IFN-γ production after ConA stimulation was significantly higher (*p* = 0.0113) in ELISA negative dogs (median: 1653 pg/mL range: 0–5318.20 pg/mL; 25th–75th percentile: 766.6–2768.1 pg/mL) when compared to ELISA positive dogs (median: 140.9 pg/mL; range: 35.6–213.8 pg/mL; 25th–75th percentile: 35.6–213.8 pg/mL). Conversely, the difference was not significant in cats. No more differences were found in IFN-γ production after ConA stimulation according to IFAT titer, parasite load, LSA-IFN-γ, health status, and the presence of clinical signs compatible with leishmaniosis. The levels of ConA-IFN-γ were statistically higher compared to those obtained after LSA stimulation in dogs (Table 2).

### 3.6. Overall Positivity

Dog and cat overall positivity is reported in Table 2.

### 3.7. FIV

Ten cats were antibody-positive to FIV (9.2%). All FIV-positive cats were adult and more frequently male cats (8/37, 21.6%) compared to female positive cats (1/63, 1.6%), (*p* = 0.0013; OR = 17.1; 95% CI = 2.309–192.3). All FIV-positive cats were *L. infantum* ELISA or PCR-negative and only three cats were IFAT-positive (titer 1:80) but no significant difference was found between FIV antibody-positive and negative cats about *L. infantum* positivity at IFAT. Among the ten FIV-positive cats, LSA and ConA-IFN-γ production were evaluated in only four cats. Production of IFN-γ was observed in only one cat after LSA stimulation and in all four cats after ConA stimulation.

## 4. Discussion

This cross-sectional study compared for the first time, the adaptive humoral and cell-mediated immune response to *L. infantum* of cats and dogs naturally exposed to the parasite in an endemic area (South of Spain) and sampled during the same sand fly season. In the current study, the level of dog’s parasite-specific immune response was generally higher compared to cats. In particular, although the proportions of antibody-positive individuals were similar (24.3% of cats and 27.1% of dogs), anti-*L. infantum* antibody levels were significantly higher in dogs than in cats for both IFAT and ELISA. Moreover, although the difference was not significant, 20% of dogs produced IFN-γ compared to 16% of cats, and the median concentration of IFN-γ produced by dogs was up to four times higher compared to cats. At the same time, parasite DNA was found in 11.9% of dog and 8.3% of cat blood samples studied. Similarly, Otranto et al. (2017) found a higher prevalence of antibody and/or PCR positivity in dogs compared to cats examined in the Eolian islands [13]. In Israel, in a hyperendemic focus detected in an animal shelter, dogs were positive for *L. infantum* direct analysis more frequently than cats and they also displayed a higher parasite load. On the other hand, cats showed antibody prevalence (ELISA test) and levels of antibody positivity higher than dogs [40].

To date, few field studies investigated the role of cellular immunity against *L. infantum* using IFN-γ assays in dogs [22,23,25,41], and only once it was performed in cats [4]. In this study, all IFN-γ producer dogs and cats had a negative or low positive antibody status without parasitemia and conversely, animals with high parasite loads and/or antibody levels did not produce IFN-γ after LSA stimulation. This finding confirms what we previously documented in cats from Catalonia (Spain) and Sicily (Italy) [4], and was already reported in several studies performed in dogs where strong humoral immune response and high blood parasitemia, were associated with the lack of cellular-specific anti-*L. infantum* IFN-γ production and progress to more severe disease [22,23,41,42].

Another important finding of this study is the occurrence of PCR-positive but antibody and IFN-γ negative animals. This pattern can be explained as an early phase of infection that occurred during the sand fly season when we performed the study and adaptive immune response was not yet elicited, or the infection did not progress in these hosts as it was already reported in dogs [43]. At present, this is only postulated in cats as longitudinal field studies are lacking.

In summary, the immunopathogenesis of *L. infantum* infection in cats shows similarities when compared to canine response, and cats can mount a specific Th1 immune response against *L. infantum.* Cats with high antibody levels or with positive blood PCR are less able to produce specific IFN-γ [4]. However, cats seem to have a lower level of both humoral and cell-mediated immune response compared to dogs [4]. Unfortunately, we were able to evaluate only a single cytokine, and to better evaluate differences between the two host species other markers of innate and adaptive immunity could be evaluated.

A high prevalence of exposure to *L. infantum* was found in both species, with 30.5% of shelter dogs and 30% of stray cats antibody-positive and/or blood PCR positive. Importantly, the rate of exposure was higher when *L. infantum-*specific IFN-γ production was measured in both dogs (44.8%) and cats (35.1%). In fact, some dogs (n = 5) and cats (n = 5) negative for anti-*L. infantum* antibodies and parasite DNA in the blood produced *L. infantum* specific IFN-γ in the WBA. Interferon-γ is a marker of Th1 immune response, which is prevalent in “resistant” dogs. Some of these dogs do not produce antibodies or have very low levels (sometimes under the cut-off value). However, parasite DNA could be detected in some of these individuals when other tissues are tested, such as bone marrow and lymph nodes [36,44]. We may assume that cats have immunological patterns similar to those of dogs in case of exposure to *Li* [4]. Therefore, when multiple tests are used in endemic areas to assess the prevalence of exposure and infection to *Li* in dogs and cats, the combination of results from parasitological (PCR) and multiple immunological markers (specific antibodies and IFN-γ) increase diagnostic sensitivity. This result confirms the endemicity of leishmaniosis in the investigated region and put in evidence that more than one-third of stray cats studied had contact with or were infected by *L. infantum*. The high prevalence found in cats from the Córdoba area is very similar to those found in the previous study we conducted in Sicily (35%) and Catalonia (36%) [4].

Stray cats can potentially be more exposed than owned cats due to the outdoor lifestyle and the lack of preventive sand fly bite treatment and for these reasons their exposure to *L. infantum* was studied in different Spanish areas and other European countries (Table 4) [45,46,47,48,49,50,51,52,53,54,55,56]. Results from different studies are difficult to compare because of analytical differences in the test used, such as the cut-off of serological tests, and the sample type for PCR test, but also the differences regarding the geographic area, the sample size, and the season of sampling [57].

Agreement between the two serological methods used was found in dogs but not in cats, and this discrepancy is not unusual and has already been reported by us and also in other studies [4,10,53].

Retroviral infections caused by FIV and FeLV have been associated in some studies with *L. infantum* infection [45,49,58,59,60,61]. Conversely, other studies did not find a statistically significant association between FIV and *L. infantum* infection in cats [40,62,63]. In this study, only ten cats were found FIV antibody-positive and all of them were blood PCR negative and antibody-negative for *Leishmania* ELISA, while three were border-line positive at IFAT. We did not find significant differences in IFN-γ production after stimulation with LSA in FIV-positive cats, but the sample size of examined cats was small and we had the opportunity to evaluate IFN-γ production in only four FIV-positive cats, and three of them did not produce IFN-γ. On the other hand, in a previous study, we tested a FIV and FeLV sick cat with positive PCR to *L. infantum* which had also high levels of antibodies and production of LSA-IFN-γ [4]. No definitive assumption is therefore possible about the role of FIV in the immunopathogenesis of *L. infantum* infection in cats and more extensive investigations are needed [64].

In an urban context, shelter dogs play a crucial role in the maintenance of *L. infantum* infection endemicity. Furthermore, the lack of preventive treatments due to financial restrictions and particularly long sheltered periods that became years, increased the endemicity as was observed in this study [55,65,66]. The current survey indicates that stray cats are exposed to *L. infantum* infection similarly to dogs in endemic areas. In light of some considerations, cats can contribute to endemicity since they are exposed to sand fly bites as much as dogs [4,13,15,28,67,68]; infection often develops subclinically with a high number of cats with positive blood PCR or positive blood culture but with no evidence of clinical signs [4,69] causing an impossibility to strictly control their parasitological status and prevent sand fly bites.

This study presents some limitations that could influence results and preclude a more robust interpretation. It was a cross-sectional study, and the number of dogs studied was small, as well as the number of animals tested for IFN-γ production. Another limitation of this study was that most dogs studied were Greyhound, and we cannot exclude breed differences in the levels of IFN-γ production. Additionally, we could not make a full clinical and clinicopathological evaluation of enrolled dogs and cats. Moreover, parasite load was measured using blood samples; thus, some infected animals were possibly missed since the detection of *L. infantum* DNA in dogs and probably in cats too, from blood is less sensitive than from other tissues [1,2]. As concerning cats, we enrolled stray cats captured for a trap-neuter-release program that mostly involves young female cats making it difficult to precisely age these cats. As a consequence, the cats under study were younger compared to shelter dogs and a higher prevalence of female cats compared to female dogs was also observed. Dogs and cats differed also in their reproductive status as all dogs were castrated and all cats entire, and we do not know if this difference could influence their immune response. The age-related bias is particularly important when the rate of positivity among the two species is compared because stray cats were exposed to fewer transmission seasons compared to dogs. However, despite the young age, a high rate of positivity was found in stray cats from Córdoba.

## 5. Conclusions

In conclusion, this study proved that in an endemic area, a consistent proportion of stray cats are exposed to *L. infantum* and are able to mount a specific anti-*L. infantum* cell-mediated immune response which is similar to dogs. However, a higher proportion of kennel dogs had contact with the parasite. More extensive clinical investigations evaluating immunological markers of the innate and adaptive immune response are needed to better understand the susceptibility of cats to develop the severe disease compared to dogs and the role of retroviral infections.

## Figures and Tables

**Table 1:** Demographic data of enrolled animals and *p* values for significant differences between dogs and cats.

Variable	Cat N (%) Dog N (%)		*p*	OR	95% CI
Sex *			0.0466	2.018	1.044–3.765
Female	63 (57.8)	27 (45.8)			
Male	37 (34)	32 (54.2)			
Unreported	9 (8.2)	0			
Age class *			0.0053	4.474	1.515–12.39
Young	26 (23.8)	4 (6.8)			
Adult and old	77 (70.6)	53 (89.8)			
Unknown	6 (5.5)	2 (3.4)			
Breed			na	na	na
Cat					
DSH	109	na			
Dog					
Crossbreed	na	20 (33.9)			
AWCRHD	na	1 (1.7)			
JRT	na	1 (1.7)			
Greyhound	na	32 (54.2)			
German shepherd	na	1 (1.7)			
Podenco	na	2 (3.4)			
Shar-pei	na	1 (1.7)			
Pointer	na	1 (1.7)			

DSH = Domestic Shorthair; AWCRHD = Andalusian Wine-Cellar Rat-Hunting Dog; JRT = Jack Russell terrier; na = not applicable; * = significant difference; 95% CI = 95% confidence interval; OR = odds ratio.

**Table 2:** Number and percentage values of dogs and cats positive to different diagnostic tests for *L. infantum*, median, 25th and 75th percentiles and range of results for different tests.

Test and Species	N (%)	Median	25th–75th Percentile	Range	*p*		OR	95% CI
					MWT	FET		
ELISA and/or IFAT					na	ns	na	na
Cats	26/107 (24.3)	na	na	na				
Dogs	16/59 (27.1)	na	na	na				
ELISA (EU) *					0.0035	0.0020	7.075	1.907–24.53
Cats	3/107 (2.8)	14.75	13.69–19.57	13.69–19.57				
Dogs	10/59 (17)	297.9	40.43–400	36.51–400				
IFAT (Titer) *					<0.0001			
Cats	24/109 (22)	1:80	1:80–1:160	1:80–1:320				
Dogs	16/59 (27.1)	1:640	1:160–1:5120	1:160–1:10,280				
PCR (am/mL)								
Cats	9/109 (8.3)	60	47.5–72.5	30–75				
Dogs	7/59 (11.9)	82	10–250	5–440				
LSA-IFN-γ (pg/mL)								
Cats §	9/57 (15.8)	45.5	40.1–79.5	33.5–172.3				
Dogs #	6/29 (20.7)	176.2	72.2–2465.1	70.6–3241				
ConA-IFN-γ (pg/mL)								
Cats §	55/57 (96.5)	1531.5	203.6–4295	35.1–9492.6				
Dogs #	28/29 (96.5)	2048.5	593.8–2534	69.5–5319				
Overall positivity								
Cats	32/107 (30) ^							
	20/57 (35.1) ^~^	na	na	na				
Dogs	18/59 (30.5) ^							
	13/29 (44.8) ^~^	na	na	na				

ELISA = enzyme-linked immunosorbent assay; IFAT = immunofluorescence antibody test; EU = ELISA unit; PCR = polymerase chain reaction; LSA-IFN-γ = *L. infantum* specific interferon-γ concentration; na = not applicable; FET = Fisher’s Exact test; MWT = Mann Whitney’s test; * = significant difference; OR = odds ratio; 95% CI = 95% confidence interval; ^ = overall positivity including individuals positive to at least one of the following tests: IFAT and/or ELISA and/or PCR; ^~^ = overall positivity including individuals positive to at least one of the following tests: IFAT and/or ELISA and/or PCR and/or LSA-IFN- γ; am = amastigotes. # = the levels of ConA-IFN-γ were statistically higher compared to those obtained after LSA stimulation in dogs (*p* < 0.0001, ConA-IFN-γ median: 1515 pg/mL, range: 35.6-5319 pg/mL, 25th–75th percentile: 476.4-2651 pg/mL, LSA-IFN-γ median: 20.39 pg/mL, range: 0–3241 pg/mL, 25th–75th percentile: 1.5–68.33 pg/mL). § = the levels of ConA-IFN-γ were statistically higher compared to those obtained after LSA stimulation in cats (*p* < 0.0001, ConA-IFN-γ median: 951 pg/mL, range: 0–9493 pg/mL, 25th 75th percentile: 140.5–3378 pg/mL, LSA-IFN-γ median: 0 pg/mL, range: 0–172.3 pg/mL, 25th–75th percentile: 0–13.06 pg/mL).

**Table 3:** Description of the number of IFN-γ producer and not producer dogs and cats positive at IFAT, ELISA, IFAT + ELISA or PCR with description of the IFAT titers and ELISA units (EU).

LSA-IFN-γ	IFAT (N, Titer)	ELISA	IFAT + ELISA	PCR
Producer dogs: 6	1 (1:160)	0	0	0
Producer cats: 9	3 (1:80)	1 (19.6 EU)	0	0
Not producer dogs: 23	5 (1:160–1:10,280)	3 (41.3–400 EU)	3	4
Not producer cats: 48	8 (1:80–1:320)	0	0	3

**Table 4:** Studies evaluating *Leishmania infantum* antibody and PCR prevalences in cats from European endemic areas.

Reference	Country (Region or City)	Lifestyle	Number of Cats Studied	Percentage of Positivity to *Leishmania infantum* by Serological and PCR Tests			
				IFAT (Cut off)	ELISA (Serum Dilution)	Western Blot (Serum Dilution)	PCR (Tissue)
Alcover et al., 2021 [45]	Spain (Zaragoza)	Stray	180	2.2 (1:20)	2.8% (1:200)	14.5 (1:200)	5.6 (blood)
Miró et al., 2014 [46]	Spain (Madrid)	Stray	346	3.2 (1:100)	n.p.	n.p.	0 (blood)
Montoya et al., 2018 [47]	Spain (Madrid, Toledo, Guadalajara, Cuenca)	Stray	632	4.8 (1:100)	n.p	n.p.	0 (blood) 0 (skin)
Millán et al., 2011 [48]	Spain (Mallorca)	Stray	86	n.p	n.p.	16 (1:10)	26 (blood and/or spleen)
Spada et al., 2013 [49]	Italy (Milan)	Stray	233	25.3 (1:40)	n.p.	n.p.	0 (blood)
Spada et al., 2016 [50]	Italy (Milan)	Stray	90	30 (1:40)	n.p.	n.p.	1.1 (blood) 1.1 (lymph node) 0 (conjunctival swabs)
Spada et al., 2020 [51]	Italy (Milan)	Stray	117	4.9 (1:80)	n.p.	n.p.	0 (blood) 4.3 (lymph node) 0 (conjunctival swabs)
Morganti et al., 2019 [52]	Italy (Umbria, Tuscany, Marche)	Cattery and colony cats	286	10.8 (1:40)	n.p	n.p.	0% (blood) 15.7% (conjunctival swabs)
Duarte et al., 2010 [53]	Portugal (Lisbon)	Stray	231	0.6 (1:40)	n.p.	n.p.	n.p.
Maia et al., 2014 [54]	Portugal (Lisbon, Setúbal, Faro)	Stray Owned indoors and outodoors	329 320	n.p.	n.p	n.p.	8.6 (blood)
Diakou et al., 2009 [55]	Greece (Thessaloniki)	Stray	284	n.p.	3.87% (n.a.)	n.p.	n.p.
Diakou et al., 2017 [56]	Greece (Crete, Mykonos, Skopelos, Athens)	Stray and free-roaming	148	6.1 (1:80)	n.p.	n.p.	6.1 (blood)

n.p.: not performed; n.a.: not available.

## Data Availability

Not applicable.
